# Dichloridobis(methanol-κ*O*)[*cis*-(±)-2,4,5-tris­(pyridin-2-yl)-2-imidazoline-κ^3^ *N* ^2^,*N* ^3^,*N* ^4^]ytterbium(III) chloride

**DOI:** 10.1107/S1600536812022052

**Published:** 2012-05-26

**Authors:** Alberto Baez-Castro, Herbert Höpfl, Miguel Parra-Hake, Adriana Cruz-Enriquez, Jose J. Campos-Gaxiola

**Affiliations:** a Facultad de Ingenieria Mochis, Universidad Autonoma de Sinaloa, Fuente Poseidon y Prol. A. Flores S/N, CP 81223, C.U. Los Mochis, Sinaloa, Mexico; b Centro de Investigaciones Quimicas, Universidad Autonoma del Estado de Morelos, Av. Universidad 1001, CP 62210, Cuernavaca, Morelos, Mexico; c Centro de Graduados del Instituto Tecnologico de Tijuana, Blvd. Industrial S/N, Col. Otay, CP 22500, Tijuana, BC, Mexico

## Abstract

In the crystal structure of the title complex, [YbCl_2_(C_18_H_15_N_5_)(CH_3_OH)_2_]Cl, the pseudo-penta­gonal–bipyramidal coordination geometry of the Yb^III^ cation is composed of three N atoms from one *cis*-(±)-2,4,5-tris­(pyridin-2-yl)imidazoline (H*L*) ligand, two O atoms from two methanol mol­ecules and two Cl^−^ anions. Chains are formed along [010] through N—H⋯Cl, O—H⋯Cl and O—H⋯N hydrogen bonds.

## Related literature

For background to the synthesis of H*L*, see: Later *et al.* (1998 ▶); Fernandes *et al.* (2007 ▶). For metal complexes with H*L*, see: Parra-Hake *et al.* (2000 ▶); Campos-Gaxiola *et al.* (2007 ▶, 2008 ▶, 2010 ▶). For related Yb (III) complexes, see: Li *et al.* (2007 ▶); Xu *et al.* (2009 ▶); Stojanovic *et al.* (2010 ▶); Okawara *et al.* (2012 ▶). For potential applications of polypyridyl chelating ligands in magnetic, electronic and luminescent devices, see: Freidzon *et al.* (2011 ▶); Maynard *et al.* (2009 ▶); Thomas *et al.* (2012 ▶).
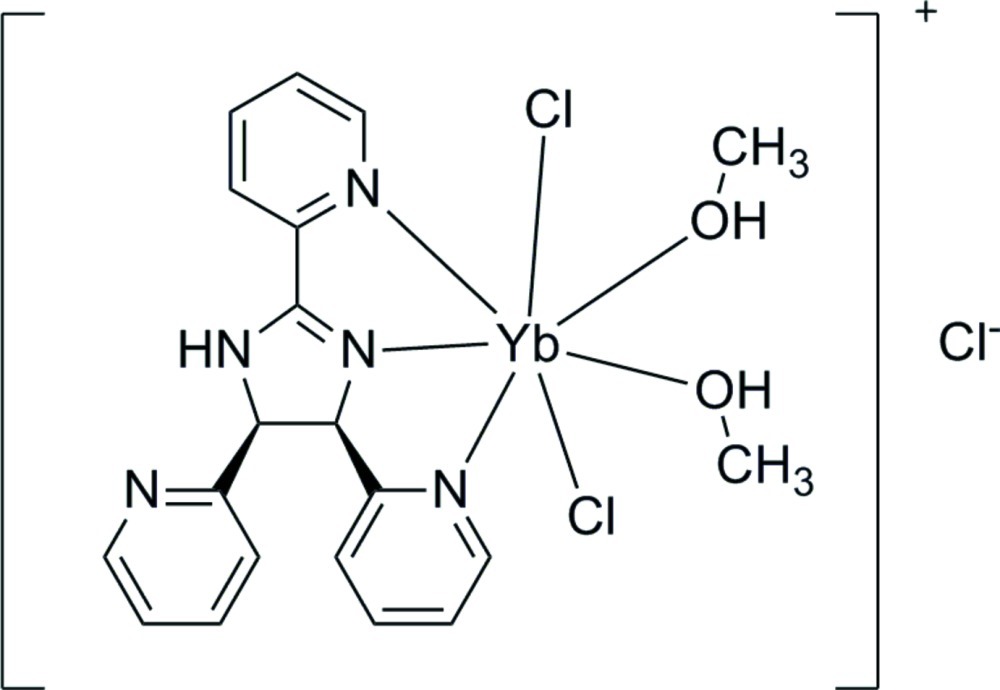


## Experimental

### 

#### Crystal data

[YbCl_2_(C_18_H_15_N_5_)(CH_4_O)_2_]Cl
*M* *_r_* = 644.82
Triclinic, 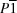

*a* = 9.2401 (13) Å
*b* = 9.8390 (14) Å
*c* = 13.3765 (19) Å
α = 99.978 (2)°
β = 94.616 (2)°
γ = 92.145 (2)°
*V* = 1192.2 (3) Å^3^
*Z* = 2
Mo *K*α radiation
μ = 4.29 mm^−1^
*T* = 293 K
0.34 × 0.29 × 0.24 mm

#### Data collection

Bruker APEX CCD area-detector diffractometer
Absorption correction: multi-scan (*SADABS*; Sheldrick, 1996 ▶) *T*_min_ = 0.32, *T*_max_ = 0.43
11582 measured reflections
4178 independent reflections
3995 reflections with *I* > 2σ(*I*)
*R*_int_ = 0.031

#### Refinement

*R*[*F* ^2^ > 2σ(*F* ^2^)] = 0.035
*wR*(*F* ^2^) = 0.091
*S* = 1.08
4178 reflections
292 parameters
3 restraints
H atoms treated by a mixture of independent and constrained refinement
Δρ_max_ = 1.10 e Å^−3^
Δρ_min_ = −1.35 e Å^−3^

### 

Data collection: *SMART* (Bruker, 2000 ▶); cell refinement: *SAINT-Plus-NT* (Bruker 2001 ▶); data reduction: *SAINT-Plus-NT*; program(s) used to solve structure: *SHELXTL-NT* (Sheldrick, 2008 ▶); program(s) used to refine structure: *SHELXTL-NT*; molecular graphics: *SHELXTL-NT*; software used to prepare material for publication: *publCIF* (Westrip, 2010 ▶).

## Supplementary Material

Crystal structure: contains datablock(s) I, global. DOI: 10.1107/S1600536812022052/fj2554sup1.cif

Structure factors: contains datablock(s) I. DOI: 10.1107/S1600536812022052/fj2554Isup2.hkl

Additional supplementary materials:  crystallographic information; 3D view; checkCIF report

## Figures and Tables

**Table 1:** Selected bond lengths (Å)

Yb1—Cl1	2.5220 (19)
Yb1—Cl2	2.5834 (18)
Yb1—N1	2.268 (5)
Yb1—N3	2.579 (5)
Yb1—N4	2.556 (6)
Yb1—O1	2.289 (5)
Yb1—O2	2.287 (5)

**Table 2:** Hydrogen-bond geometry (Å, °)

*D*—H⋯*A*	*D*—H	H⋯*A*	*D*⋯*A*	*D*—H⋯*A*
O1—H10′⋯N5^i^	0.84	1.86	2.681 (3)	164
O2—H20′⋯Cl3^i^	0.84	2.19	2.956 (3)	152
N2—H2′⋯Cl3	0.86	2.25	3.096 (3)	167

Symmetry code: (i) 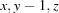.

**Fig. 1.**
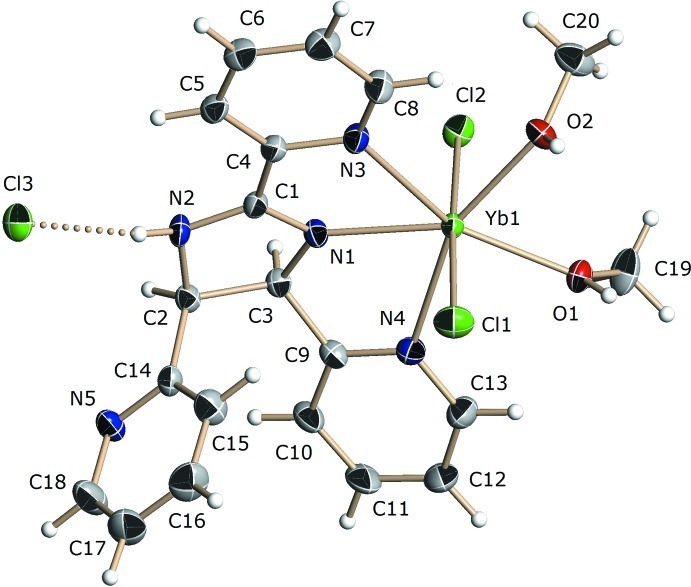
Perspective view of the molecular structure of the title compound, with atom labels and 30% probability displacement ellipsoids.

**Fig. 2.**
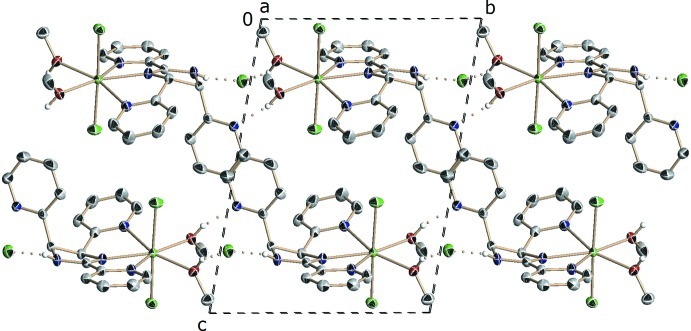
Perspective view of a fragment of the linear supramolecular chain along [010] with the N—H···Cl, O—H···Cl and O—H···N hydrogen bonds shown as dashed lines.

## supplementary crystallographic information

### Comment

The synthesis and characterization of lanthanide complexes supported by polypiridyl chelating ligands has attracted continuous interest, due to the potential application of these compounds in magnetic, electronic and luminescent devices (Maynard *et al.*, 2009; Freidzon *et al.*, 2011; Thomas *et al.*, 2012). A handful of transition metal complexes based on the HL ligand have been prepared (Parra-Hake *et al.* Campos-Gaxiola *et al.*, 2007, 2008 and 2010), but to date there are no reports on complexes containing rare earth elements. Herein, we report on the structure of a mononuclear Yb^III^ complex (Scheme 1), which was synthesized by reaction of YbCl_3_^.^6H_2_O with *cis*-(±)-2,4,5-tri(2-pyridyl)imidazoline at room temperature in methanol.

In the title complex, the central Yb^III^ ion is seven-coordinated by three nitrogen atoms from one tridentate chelate ligand, two oxygen atoms from two methanol molecules and two Cl^-^ anions [Yb—N, 2.268 (5)–2.579 (5) Å; Yb—O 2.287 (4)–2.289 (4) Å; Yb—Cl 2.5219 (18)–2.5834 (17) Å (Table 1)] and displays a pseudo-pentagonal-bipyramidal geometry (Fig. 1). In the crystal structure the complex molecules are linked through N—H···Cl, O—H···Cl and O—H···N hydrogen bonds to form linear supramolecular chains along [010] (Table 2, Fig 2).

### Experimental

A mixture of *cis*-(±)-2,4,5-tri(2-pyridyl)imidazoline (HL) (0.05 g, 0.166 mmol) and YbCl_3_^.^6H_2_O (0.063 g, 0.166 mmol) dissolved in methanol (3 ml) was stirred for 2 h at room temperature to give a colorless solution. The product was crystallized at room temperature by gas phase diffusion of diethyl ether into the reaction mixture, providing colorless crystals which were dried under vacuo. Yield: 71%. IR (KBr): 3320, 3060, 3155, 2894, 1631, 1592, 1471, 1437, 1338, 1280, 1260, 1162, 1007 and 691 cm^-1^. TGA Calcd for 2CH_3_OH: 9.94. Found: 8.76% (30–200 °C).

### Refinement

C—H atoms were positioned geometrically and constrained using the riding-model approximation [*C*—H_aryl_ = 0.93 Å, *U*_iso_(H_aryl_) = 1.2 *U*_eq_(C)]. The coordinates of the O—H and N—H hydrogen atoms were refined with distances restraints: O—H = 0.840±0.001 Å, N—H = 0.860±0.001 Å and [*U*_iso_(H) = 1.5 *U*_eq_(O,*N*)].

### Crystal data

**Table:**

[YbCl_2_(C_18_H_15_N_5_)(CH_4_O)_2_]Cl	*Z* = 2
*M**_r_* = 644.82	*F*(000) = 630
Triclinic, *P*1	*D*_x_ = 1.796 Mg m^−^^3^
Hall symbol: -P 1	Mo *K*α radiation, λ = 0.71073 Å
*a* = 9.2401 (13) Å	Cell parameters from 7905 reflections
*b* = 9.8390 (14) Å	θ = 2.2–28.3°
*c* = 13.3765 (19) Å	µ = 4.29 mm^−^^1^
α = 99.978 (2)°	*T* = 293 K
β = 94.616 (2)°	Rectangular prism, colorless
γ = 92.145 (2)°	0.34 × 0.29 × 0.24 mm
*V* = 1192.2 (3) Å^3^	

### Data collection

**Table:**

Bruker APEX CCD area-detector diffractometer	4178 independent reflections
Radiation source: fine-focus sealed tube	3995 reflections with *I* > 2σ(*I*)
Graphite monochromator	*R*_int_ = 0.031
Detector resolution: 8.3 pixels mm^-1^	θ_max_ = 25.0°, θ_min_ = 1.6°
phi and ω scans	*h* = −10→10
Absorption correction: multi-scan (*SADABS*; Sheldrick, 1996)	*k* = −11→11
*T*_min_ = 0.32, *T*_max_ = 0.43	*l* = −15→15
11582 measured reflections	

### Refinement

**Table:**

Refinement on *F*^2^	Primary atom site location: structure-invariant direct methods
Least-squares matrix: full	Secondary atom site location: difference Fourier map
*R*[*F*^2^ > 2σ(*F*^2^)] = 0.035	Hydrogen site location: inferred from neighbouring sites
*wR*(*F*^2^) = 0.091	H atoms treated by a mixture of independent and constrained refinement
*S* = 1.08	*w* = 1/[σ^2^(*F*_o_^2^) + (0.0428*P*)^2^ + 3.5218*P*] where *P* = (*F*_o_^2^ + 2*F*_c_^2^)/3
4178 reflections	(Δ/σ)_max_ < 0.001
292 parameters	Δρ_max_ = 1.10 e Å^−^^3^
3 restraints	Δρ_min_ = −1.35 e Å^−^^3^

### Special details

**Table:**

Geometry. All e.s.d.'s (except the e.s.d. in the dihedral angle between two l.s. planes) are estimated using the full covariance matrix. The cell e.s.d.'s are taken into account individually in the estimation of e.s.d.'s in distances, angles and torsion angles; correlations between e.s.d.'s in cell parameters are only used when they are defined by crystal symmetry. An approximate (isotropic) treatment of cell e.s.d.'s is used for estimating e.s.d.'s involving l.s. planes.
Refinement. Refinement of *F*^2^ against ALL reflections. The weighted *R*-factor *wR* and goodness of fit *S* are based on *F*^2^, conventional *R*-factors *R* are based on *F*, with *F* set to zero for negative *F*^2^. The threshold expression of *F*^2^ > σ(*F*^2^) is used only for calculating *R*-factors(gt) *etc*. and is not relevant to the choice of reflections for refinement. *R*-factors based on *F*^2^ are statistically about twice as large as those based on *F*, and *R*- factors based on ALL data will be even larger.

### Fractional atomic coordinates and isotropic or equivalent isotropic displacement parameters (Å^2^)

**Table:**

	*x*	*y*	*z*	*U*_iso_*/*U*_eq_	
Yb1	0.21176 (3)	0.30381 (2)	0.204195 (19)	0.02770 (10)	
Cl1	0.3560 (2)	0.3299 (2)	0.37530 (14)	0.0560 (5)	
Cl2	0.05316 (18)	0.26855 (19)	0.03273 (13)	0.0444 (4)	
Cl3	0.57461 (18)	0.96378 (19)	0.21144 (15)	0.0486 (4)	
N1	0.2212 (5)	0.5280 (5)	0.1839 (4)	0.0333 (12)	
N2	0.3278 (6)	0.7350 (5)	0.1816 (5)	0.0383 (13)	
H2'	0.400 (5)	0.794 (6)	0.199 (6)	0.057*	
N3	0.4539 (6)	0.3892 (5)	0.1471 (4)	0.0381 (13)	
N4	0.0248 (6)	0.4541 (6)	0.2944 (4)	0.0406 (13)	
N5	0.1688 (7)	0.9593 (6)	0.3636 (4)	0.0458 (14)	
O1	0.0623 (5)	0.1401 (5)	0.2518 (4)	0.0382 (10)	
H10'	0.099 (8)	0.097 (7)	0.296 (4)	0.057*	
O2	0.3170 (5)	0.1033 (5)	0.1423 (4)	0.0411 (11)	
H20'	0.380 (6)	0.076 (8)	0.182 (5)	0.062*	
C1	0.3387 (7)	0.5981 (6)	0.1703 (5)	0.0343 (14)	
C2	0.1917 (7)	0.7706 (6)	0.2265 (5)	0.0339 (14)	
H2	0.1415	0.8351	0.1894	0.041*	
C3	0.1082 (6)	0.6265 (6)	0.2014 (5)	0.0331 (14)	
H3	0.0496	0.6223	0.1364	0.040*	
C4	0.4686 (7)	0.5258 (6)	0.1447 (5)	0.0343 (14)	
C5	0.5934 (7)	0.5880 (7)	0.1193 (5)	0.0394 (15)	
H5	0.6006	0.6827	0.1198	0.047*	
C6	0.7078 (7)	0.5061 (8)	0.0931 (5)	0.0443 (17)	
H6	0.7925	0.5443	0.0739	0.053*	
C7	0.6944 (8)	0.3692 (8)	0.0961 (6)	0.0490 (18)	
H7	0.7709	0.3131	0.0795	0.059*	
C8	0.5684 (7)	0.3130 (8)	0.1235 (6)	0.0476 (18)	
H8	0.5619	0.2190	0.1258	0.057*	
C9	0.0099 (7)	0.5825 (7)	0.2750 (5)	0.0362 (14)	
C10	−0.0956 (8)	0.6658 (7)	0.3153 (6)	0.0475 (18)	
H10	−0.1044	0.7539	0.2998	0.057*	
C11	−0.1872 (9)	0.6178 (8)	0.3785 (7)	0.061 (2)	
H11	−0.2592	0.6727	0.4058	0.073*	
C12	−0.1719 (10)	0.4893 (9)	0.4010 (7)	0.066 (2)	
H12	−0.2320	0.4550	0.4444	0.079*	
C13	−0.0643 (9)	0.4108 (8)	0.3574 (6)	0.057 (2)	
H13	−0.0535	0.3230	0.3730	0.068*	
C14	0.2201 (7)	0.8352 (7)	0.3373 (5)	0.0366 (14)	
C15	0.2982 (9)	0.7722 (8)	0.4064 (6)	0.0537 (19)	
H15	0.3318	0.6847	0.3863	0.064*	
C16	0.3266 (10)	0.8398 (10)	0.5062 (6)	0.068 (2)	
H16	0.3800	0.7994	0.5541	0.082*	
C17	0.2734 (11)	0.9683 (10)	0.5321 (6)	0.072 (3)	
H17	0.2910	1.0173	0.5982	0.087*	
C18	0.1945 (12)	1.0233 (9)	0.4596 (6)	0.072 (3)	
H18	0.1571	1.1096	0.4783	0.087*	
C19	−0.0775 (8)	0.0814 (9)	0.2119 (7)	0.064 (2)	
H19A	−0.1374	0.1524	0.1938	0.096*	
H19B	−0.1211	0.0390	0.2625	0.096*	
H19C	−0.0684	0.0128	0.1526	0.096*	
C20	0.2876 (9)	0.0171 (8)	0.0451 (6)	0.060 (2)	
H20A	0.1852	−0.0063	0.0328	0.090*	
H20B	0.3396	−0.0658	0.0437	0.090*	
H20C	0.3183	0.0654	−0.0067	0.090*	

### Atomic displacement parameters (Å^2^)

**Table:**

	*U*^11^	*U*^22^	*U*^33^	*U*^12^	*U*^13^	*U*^23^
Yb1	0.02727 (15)	0.02462 (15)	0.03167 (16)	0.00041 (10)	0.00363 (10)	0.00598 (10)
Cl1	0.0528 (11)	0.0726 (13)	0.0401 (10)	0.0067 (9)	−0.0046 (8)	0.0065 (9)
Cl2	0.0398 (9)	0.0511 (10)	0.0430 (9)	−0.0013 (7)	−0.0055 (7)	0.0154 (8)
Cl3	0.0381 (9)	0.0455 (10)	0.0607 (11)	−0.0070 (7)	−0.0054 (8)	0.0111 (8)
N1	0.030 (3)	0.027 (3)	0.044 (3)	0.002 (2)	0.006 (2)	0.006 (2)
N2	0.038 (3)	0.026 (3)	0.053 (3)	−0.003 (2)	0.007 (3)	0.013 (3)
N3	0.036 (3)	0.034 (3)	0.046 (3)	−0.001 (2)	0.007 (2)	0.010 (2)
N4	0.042 (3)	0.039 (3)	0.042 (3)	0.006 (3)	0.010 (3)	0.007 (3)
N5	0.065 (4)	0.033 (3)	0.037 (3)	0.006 (3)	−0.006 (3)	0.003 (2)
O1	0.035 (2)	0.036 (3)	0.045 (3)	0.0003 (19)	0.000 (2)	0.016 (2)
O2	0.041 (3)	0.035 (3)	0.043 (3)	0.005 (2)	−0.002 (2)	−0.004 (2)
C1	0.040 (4)	0.032 (3)	0.033 (3)	−0.002 (3)	0.004 (3)	0.009 (3)
C2	0.039 (3)	0.029 (3)	0.035 (3)	0.006 (3)	0.000 (3)	0.009 (3)
C3	0.030 (3)	0.031 (3)	0.038 (3)	0.001 (3)	0.000 (3)	0.006 (3)
C4	0.035 (3)	0.033 (3)	0.035 (3)	−0.001 (3)	0.004 (3)	0.006 (3)
C5	0.036 (4)	0.042 (4)	0.039 (4)	−0.007 (3)	0.003 (3)	0.007 (3)
C6	0.027 (3)	0.057 (5)	0.048 (4)	−0.007 (3)	0.002 (3)	0.009 (3)
C7	0.037 (4)	0.058 (5)	0.055 (5)	0.011 (3)	0.010 (3)	0.014 (4)
C8	0.041 (4)	0.039 (4)	0.067 (5)	0.010 (3)	0.016 (3)	0.014 (4)
C9	0.030 (3)	0.035 (4)	0.041 (4)	0.002 (3)	0.001 (3)	−0.001 (3)
C10	0.043 (4)	0.038 (4)	0.059 (5)	0.005 (3)	0.012 (3)	−0.003 (3)
C11	0.058 (5)	0.051 (5)	0.071 (6)	0.009 (4)	0.033 (4)	−0.007 (4)
C12	0.073 (6)	0.058 (5)	0.071 (6)	−0.001 (4)	0.045 (5)	0.007 (4)
C13	0.065 (5)	0.049 (5)	0.062 (5)	0.008 (4)	0.025 (4)	0.018 (4)
C14	0.040 (4)	0.032 (3)	0.038 (4)	−0.004 (3)	−0.003 (3)	0.010 (3)
C15	0.064 (5)	0.047 (4)	0.050 (4)	0.012 (4)	−0.007 (4)	0.013 (4)
C16	0.079 (6)	0.079 (6)	0.048 (5)	0.003 (5)	−0.013 (4)	0.024 (4)
C17	0.107 (8)	0.065 (6)	0.040 (5)	−0.004 (5)	−0.008 (5)	0.004 (4)
C18	0.128 (9)	0.043 (5)	0.043 (5)	0.013 (5)	−0.002 (5)	0.000 (4)
C19	0.044 (4)	0.063 (5)	0.088 (6)	−0.017 (4)	−0.010 (4)	0.036 (5)
C20	0.071 (5)	0.057 (5)	0.049 (5)	0.024 (4)	−0.003 (4)	0.001 (4)

### Geometric parameters (Å, º)

**Table:**

Yb1—Cl1	2.5220 (19)	C5—C6	1.384 (10)
Yb1—Cl2	2.5834 (18)	C5—H5	0.93
Yb1—N1	2.268 (5)	C6—C7	1.355 (11)
Yb1—N3	2.579 (5)	C6—H6	0.93
Yb1—N4	2.556 (6)	C7—C8	1.373 (10)
Yb1—O1	2.289 (5)	C7—H7	0.9301
Yb1—O2	2.287 (5)	C8—H8	0.9303
N1—C1	1.306 (8)	C9—C10	1.380 (10)
N1—C3	1.457 (8)	C10—C11	1.370 (12)
N2—C1	1.338 (8)	C10—H10	0.9302
N2—C2	1.465 (9)	C11—C12	1.359 (12)
N2—H2'	0.86 (6)	C11—H11	0.9302
N3—C4	1.352 (9)	C12—C13	1.386 (12)
N3—C8	1.346 (9)	C12—H12	0.9294
N4—C13	1.333 (10)	C13—H13	0.9303
N4—C9	1.343 (9)	C14—C15	1.373 (10)
N5—C14	1.326 (9)	C15—C16	1.384 (11)
N5—C18	1.327 (10)	C15—H15	0.9294
O1—C19	1.419 (9)	C16—C17	1.372 (14)
O1—H10'	0.84 (6)	C16—H16	0.9303
O2—C20	1.424 (10)	C17—C18	1.365 (13)
O2—H20'	0.84 (6)	C17—H17	0.9295
C1—C4	1.453 (9)	C18—H18	0.9304
C2—C14	1.506 (9)	C19—H19A	0.9601
C2—C3	1.559 (9)	C19—H19B	0.9598
C2—H2	0.9797	C19—H19C	0.9595
C3—C9	1.496 (9)	C20—H20A	0.96
C3—H3	0.9797	C20—H20B	0.9599
C4—C5	1.379 (9)	C20—H20C	0.96
			
N1—Yb1—O2	138.18 (18)	N1—C3—H3	107.27
N1—Yb1—O1	141.84 (17)	C2—C3—H3	107.27
O2—Yb1—O1	77.76 (17)	C9—C3—H3	107.29
N1—Yb1—Cl1	99.05 (14)	N3—C4—C5	123.1 (6)
O2—Yb1—Cl1	92.85 (12)	N3—C4—C1	112.6 (5)
O1—Yb1—Cl1	89.06 (12)	C5—C4—C1	124.3 (6)
N1—Yb1—N4	64.63 (18)	C4—C5—C6	118.3 (6)
O2—Yb1—N4	156.52 (18)	C4—C5—H5	120.84
O1—Yb1—N4	78.77 (17)	C6—C5—H5	120.83
Cl1—Yb1—N4	87.06 (14)	C7—C6—C5	118.9 (6)
N1—Yb1—N3	65.20 (17)	C5—C6—H6	120.5
O2—Yb1—N3	76.74 (17)	C7—C6—H6	120.57
O1—Yb1—N3	152.94 (17)	C6—C7—C8	120.3 (7)
Cl1—Yb1—N3	83.32 (13)	C6—C7—H7	119.81
N4—Yb1—N3	126.44 (17)	C8—C7—H7	119.9
N1—Yb1—Cl2	83.18 (14)	N3—C8—C7	122.3 (7)
O2—Yb1—Cl2	86.99 (12)	N3—C8—H8	118.86
O1—Yb1—Cl2	87.74 (12)	C7—C8—H8	118.87
Cl1—Yb1—Cl2	176.75 (6)	N4—C9—C10	122.1 (6)
N4—Yb1—Cl2	91.79 (13)	N4—C9—C3	115.9 (5)
N3—Yb1—Cl2	99.79 (13)	C10—C9—C3	121.9 (6)
N1—Yb1—C1	19.51 (17)	C11—C10—C9	119.5 (7)
O2—Yb1—C1	121.66 (17)	C9—C10—H10	120.28
O1—Yb1—C1	160.52 (16)	C11—C10—H10	120.24
Cl1—Yb1—C1	91.30 (12)	C12—C11—C10	119.4 (7)
N4—Yb1—C1	81.80 (17)	C10—C11—H11	120.3
N3—Yb1—C1	46.10 (16)	C12—C11—H11	120.28
Cl2—Yb1—C1	91.55 (12)	C11—C12—C13	118.2 (7)
C1—N1—C3	106.9 (5)	C11—C12—H12	120.93
C1—N1—Yb1	125.0 (4)	C13—C12—H12	120.96
C3—N1—Yb1	127.2 (4)	N4—C13—C12	123.6 (7)
C1—N2—H2'	124 (4)	N4—C13—H13	118.21
C2—N2—H2'	116 (4)	C12—C13—H13	118.1
C1—N2—C2	108.6 (5)	N5—C14—C15	121.8 (6)
C8—N3—C4	117.0 (6)	N5—C14—C2	115.8 (5)
C8—N3—Yb1	126.7 (4)	C15—C14—C2	122.4 (6)
C4—N3—Yb1	116.2 (4)	C14—C15—C16	119.7 (7)
C13—N4—C9	117.2 (6)	C14—C15—H15	120.18
C13—N4—Yb1	123.8 (5)	C16—C15—H15	120.2
C9—N4—Yb1	119.0 (4)	C17—C16—C15	117.8 (8)
C14—N5—C18	118.5 (6)	C15—C16—H16	121.05
C19—O1—Yb1	132.4 (4)	C17—C16—H16	121.09
Yb1—O1—H10'	116 (5)	C18—C17—C16	119.2 (8)
C19—O1—H10'	111 (5)	C16—C17—H17	120.4
C20—O2—Yb1	128.2 (4)	C18—C17—H17	120.42
Yb1—O2—H20'	116 (5)	N5—C18—C17	122.9 (8)
C20—O2—H20'	116 (5)	O1—C19—H19C	109.45
N1—C1—N2	115.4 (6)	H19A—C19—H19C	109.51
N1—C1—C4	119.7 (6)	H19B—C19—H19C	109.53
N2—C1—C4	124.9 (6)	O1—C19—H19B	109.44
N2—C2—C14	111.0 (5)	H19A—C19—H19B	109.48
N2—C2—C3	99.8 (5)	O1—C19—H19A	109.42
C14—C2—C3	117.1 (5)	O2—C20—H20C	109.44
N2—C2—H2	109.54	H20A—C20—H20C	109.47
C3—C2—H2	109.5	H20B—C20—H20C	109.47
C14—C2—H2	109.48	O2—C20—H20B	109.48
N1—C3—C9	109.5 (5)	H20A—C20—H20B	109.48
N1—C3—C2	104.6 (5)	O2—C20—H20A	109.48
C9—C3—C2	120.3 (5)		
			
O2—Yb1—N1—C1	−37.1 (6)	N4—Yb1—C1—N1	−27.2 (5)
O1—Yb1—N1—C1	167.7 (4)	N3—Yb1—C1—N1	166.5 (6)
Cl1—Yb1—N1—C1	67.6 (5)	C1—N2—C2—C14	104.9 (6)
N4—Yb1—N1—C1	150.0 (6)	C1—N2—C2—C3	−19.3 (6)
N3—Yb1—N1—C1	−10.7 (5)	C1—N1—C3—C9	−146.0 (5)
Cl2—Yb1—N1—C1	−114.8 (5)	Yb1—N1—C3—C9	23.8 (7)
O2—Yb1—N1—C3	154.9 (4)	C1—N1—C3—C2	−15.8 (6)
O1—Yb1—N1—C3	−0.3 (6)	Yb1—N1—C3—C2	154.0 (4)
Cl1—Yb1—N1—C3	−100.4 (5)	N2—C2—C3—N1	20.8 (6)
N4—Yb1—N1—C3	−18.1 (5)	C14—C2—C3—N1	−99.1 (6)
N3—Yb1—N1—C3	−178.7 (5)	N2—C2—C3—C9	144.2 (6)
Cl2—Yb1—N1—C3	77.2 (5)	C14—C2—C3—C9	24.4 (8)
N1—Yb1—N3—C8	−176.1 (6)	C8—N3—C4—C5	0.1 (10)
O2—Yb1—N3—C8	−13.8 (6)	Yb1—N3—C4—C5	177.6 (5)
O1—Yb1—N3—C8	6.1 (8)	C8—N3—C4—C1	179.4 (6)
Cl1—Yb1—N3—C8	80.7 (6)	Yb1—N3—C4—C1	−3.0 (7)
N4—Yb1—N3—C8	162.1 (5)	N1—C1—C4—N3	−5.8 (9)
Cl2—Yb1—N3—C8	−98.3 (6)	N2—C1—C4—N3	174.4 (6)
N1—Yb1—N3—C4	6.7 (4)	N1—C1—C4—C5	173.5 (6)
O2—Yb1—N3—C4	168.9 (5)	N2—C1—C4—C5	−6.3 (10)
O1—Yb1—N3—C4	−171.1 (4)	N3—C4—C5—C6	1.4 (10)
Cl1—Yb1—N3—C4	−96.5 (4)	C1—C4—C5—C6	−177.9 (6)
N4—Yb1—N3—C4	−15.2 (5)	C4—C5—C6—C7	−1.8 (10)
Cl2—Yb1—N3—C4	84.4 (4)	C5—C6—C7—C8	0.7 (11)
N1—Yb1—N4—C13	−173.7 (7)	C4—N3—C8—C7	−1.2 (11)
O2—Yb1—N4—C13	18.1 (9)	Yb1—N3—C8—C7	−178.4 (5)
O1—Yb1—N4—C13	17.3 (6)	C6—C7—C8—N3	0.8 (12)
Cl1—Yb1—N4—C13	−72.3 (6)	C13—N4—C9—C10	−2.0 (10)
N3—Yb1—N4—C13	−151.8 (6)	Yb1—N4—C9—C10	175.4 (5)
Cl2—Yb1—N4—C13	104.7 (6)	C13—N4—C9—C3	−178.1 (6)
N1—Yb1—N4—C9	9.0 (4)	Yb1—N4—C9—C3	−0.7 (7)
O2—Yb1—N4—C9	−159.1 (4)	N1—C3—C9—N4	−12.3 (8)
O1—Yb1—N4—C9	−159.9 (5)	C2—C3—C9—N4	−133.4 (6)
Cl1—Yb1—N4—C9	110.5 (5)	N1—C3—C9—C10	171.6 (6)
N3—Yb1—N4—C9	31.0 (6)	C2—C3—C9—C10	50.5 (9)
Cl2—Yb1—N4—C9	−72.5 (5)	N4—C9—C10—C11	1.0 (11)
N1—Yb1—O1—C19	60.5 (8)	C3—C9—C10—C11	176.9 (7)
O2—Yb1—O1—C19	−102.9 (7)	C9—C10—C11—C12	0.5 (13)
Cl1—Yb1—O1—C19	164.0 (7)	C10—C11—C12—C13	−0.9 (14)
N4—Yb1—O1—C19	76.8 (7)	C9—N4—C13—C12	1.7 (12)
N3—Yb1—O1—C19	−122.7 (7)	Yb1—N4—C13—C12	−175.6 (7)
Cl2—Yb1—O1—C19	−15.4 (7)	C11—C12—C13—N4	−0.2 (14)
N1—Yb1—O2—C20	−75.2 (7)	C18—N5—C14—C15	0.0 (11)
O1—Yb1—O2—C20	89.4 (6)	C18—N5—C14—C2	−177.9 (7)
Cl1—Yb1—O2—C20	177.9 (6)	N2—C2—C14—N5	123.6 (6)
N4—Yb1—O2—C20	88.7 (7)	C3—C2—C14—N5	−122.8 (6)
N3—Yb1—O2—C20	−99.7 (6)	N2—C2—C14—C15	−54.3 (8)
Cl2—Yb1—O2—C20	1.1 (6)	C3—C2—C14—C15	59.4 (9)
C3—N1—C1—N2	3.7 (8)	N5—C14—C15—C16	−0.9 (12)
Yb1—N1—C1—N2	−166.4 (4)	C2—C14—C15—C16	176.9 (7)
C3—N1—C1—C4	−176.1 (5)	C14—C15—C16—C17	0.5 (13)
Yb1—N1—C1—C4	13.8 (8)	C15—C16—C17—C18	0.6 (15)
C2—N2—C1—N1	11.2 (8)	C14—N5—C18—C17	1.2 (14)
C2—N2—C1—C4	−169.0 (6)	C16—C17—C18—N5	−1.5 (16)
Cl1—Yb1—C1—N1	−114.0 (5)		

### Hydrogen-bond geometry (Å, º)

**Table:**

*D*—H···*A*	*D*—H	H···*A*	*D*···*A*	*D*—H···*A*
O1—H10′···N5^i^	0.84	1.86	2.681 (3)	164
O2—H20′···Cl3^i^	0.84	2.19	2.956 (3)	152
N2—H2′···Cl3	0.86	2.25	3.096 (3)	167

Symmetry code: (i) *x*, *y*−1, *z*.
